# SNPnexus: a web server for functional annotation of human genome sequence variation (2020 update)

**DOI:** 10.1093/nar/gkaa420

**Published:** 2020-06-04

**Authors:** Jorge Oscanoa, Lavanya Sivapalan, Emanuela Gadaleta, Abu Z Dayem Ullah, Nicholas R Lemoine, Claude Chelala

**Affiliations:** Centre for Cancer Biomarkers and Biotherapeutics, Barts Cancer Institute, Queen Mary University of London, London EC1M 6BQ, UK; Centre for Cancer Biomarkers and Biotherapeutics, Barts Cancer Institute, Queen Mary University of London, London EC1M 6BQ, UK; Centre for Cancer Biomarkers and Biotherapeutics, Barts Cancer Institute, Queen Mary University of London, London EC1M 6BQ, UK; Centre for Cancer Biomarkers and Biotherapeutics, Barts Cancer Institute, Queen Mary University of London, London EC1M 6BQ, UK; Centre for Cancer Biomarkers and Biotherapeutics, Barts Cancer Institute, Queen Mary University of London, London EC1M 6BQ, UK; Centre for Cancer Biomarkers and Biotherapeutics, Barts Cancer Institute, Queen Mary University of London, London EC1M 6BQ, UK

## Abstract

SNPnexus is a web-based annotation tool for the analysis and interpretation of both known and novel sequencing variations. Since its last release, SNPnexus has received continual updates to expand the range and depth of annotations provided. SNPnexus has undergone a complete overhaul of the underlying infrastructure to accommodate faster computational times. The scope for data annotation has been substantially expanded to enhance biological interpretations of queried variants. This includes the addition of pathway analysis for the identification of enriched biological pathways and molecular processes. We have further expanded the range of user directed annotation fields available for the study of cancer sequencing data. These new additions facilitate investigations into cancer driver variants and targetable molecular alterations within input datasets. New user directed filtering options have been coupled with the addition of interactive graphical and visualization tools. These improvements streamline the analysis of variants derived from large sequencing datasets for the identification of biologically and clinically significant subsets in the data. SNPnexus is the most comprehensible web-based application currently available and these new set of updates ensures that it remains a state-of-the-art tool for researchers. SNPnexus is freely available at https://www.snp-nexus.org.

## INTRODUCTION

Current high-throughput sequencing technologies produce vast amounts of variation data. Novel tools and techniques are required to facilitate the analysis and interpretation of such data. Screening of phenotypically-relevant variants requires the integration of genomic annotation data with a broad range of other annotation categories to allow users to determine possible consequences of the queried alterations. SNPnexus was designed to meet this challenge and has been updated continually (1–3) to ensure that it remains a cutting-edge analytical tool that allows the research community to asses potential significant variants.

In response to invaluable feedback from our user community, we have improved the processing speed of SNPnexus (version 4) compared to the previous release. This new version offers significant improvements in processing time especially for large queries while allowing for new relevant annotations to be retrieved. We have also introduced a dedicated queue task manager to improve the system's ability to deal with multiple concurrent queries.

Furthermore, all data sources have been updated and new categories have been created linking variants with their biological pathways and also, using the Cancer Genome Interpreter (4), predicting alterations that may act as cancer drivers and their possible effect on treatment response. Table 1 shows a summary of all SNPnexus features present in the current version. The new software architecture streamlines the process of updating current annotations or adding new ones and we intend to continue accommodating new and advance annotations to facilitate scientific discoveries.

**Table 1.** Summary of SNPnexus features present in the current version

	Feature	SNPnexus v4
**Input queries**	Human assembly	GRCh37 / GRCh38
	Variation format	Genomic coordinates (known/unknown)
		Chromosomal region (known)
		dbSNP rs# (known)
	Supported alterations	Single based substitutions, Insertions/deletions (InDel), Block substitutions
		Multiple alterations
		IUPAC code supported
	Batch query size	up to 100 000
	Batch query format	VCF or Text file in the SNPnexus variation format (See Supplementary Table S1)
**Annotation categories**	Gene annotation systems	GRCh37 and GRCh38: Ensembl, RefSeq, UCSC, CCDS
		GRCh37: Vega, AceView, H-Invitational
		Coding: Synonymous / Nonsynonymous / Stop-gain/loss / Frame-shift / Peptide-shift
		Intronic (splice site)
		Non-coding, 5′/3′-UTR, up/down-stream
	Protein deleterious effects	SIFT (known and novel) and PolyPhen (known)
	Population data	gnomAD Exomes: AFR, AMR, EAS, FIN, NFE, OTH, SAS
		gnomAD Genomes: AFR, AMR, EAS, FIN, NFE, OTH
		1000Genomes: AFR, AMR, EAS, EUR, SAS
		HapMap: ASW, CEU, CHB, CHD, GIH, HCB, JPT, LWK, MEX, MKK, TSI, YRI
	Regulatory elements	GRCh37 and GRCh38: miRBASE, CpG islands, TarBase, microRNAs / snoRNAs / scaRNAs, Ensembl Regulatory Build, ENCODE Project, Roadmap Epigenomics
		GRCh37: Transcription factor binding sites, Vista enhancers, TargetScan
	Conservation scores	GERP++ scores, PHAST
	Disease studies	GRCh37: GAD
		GRCh37 and GRCh38: COSMIC, NHGRI-GWAS, ClinVar
	Non-coding scoring	GRCh37: fitCons, EIGEN, FATHMM GWAVA, DeepSEA, ReMM
		GRCh37 and GRCh38: CADD, FunSeq2
	Structural variations	Gain, Loss, Gain+Loss, Duplication, Deletion, Insertion, Complex, Inversion, Tandem duplication, Novel sequence insertion, Mobile element insertion, Sequence alteration
	Pathway Analysis	Reactome pathways
	Biological / Clinical Interpretation	Cancer genome interpreter
**Output**	Web format	Html, graphical
	Filters for query set	Known / Novel variant
		Minor allele global frequency
		Genomic consequence
		Predicted protein effect
		In conserved region
		Known phenotype association
		Predicted cancer driver
		Genes involved in query set
		Pathways involved in query set
	Filters per annotation	Filter per dbSNP or Variation ID in each annotation table
	Exporting options	Export per annotation or all annotations
	Exporting formats	VCF or Tab-delimited texts

## PROGRAM DESCRIPTION AND METHODS

SNPnexus is a web-based variant annotation tool that gathers datasets from different sources and allows its users to annotate, assess and, through an easy-to-use set of filters, prioritize a set of variations (single nucleotide variations, Insertions and Deletions (InDels), and block substitutions) based on different features like predicted genomic consequence (using up to 7 different gene annotation systems), level of damaging effect (using SIFT (5) and PolyPhen (6)), known phenotype association (with data from COSMIC (7) and ClinVar (8)), global population prevalence, predicted driver of cancer (using Cancer Genome Interpreter data), among others.

SNPnexus accepts queries in three different formats: genomic position (using clone, contig or chromosome as reference), chromosomal region or dbSNP identifier. Using the web interface, it is possible to input variants directly using the web controls or by pasting the query in the text area. Moreover, submitting batch queries is also possible by uploading a VCF file or a text file in the SNPnexus variant format detailed in the Supplementary Table S1.

Users should first select the human reference assembly from the two available (GRCh37 and GRCh38), and then proceed to select the desired annotations. Unlike previous versions, there is no limit to the number of annotations that can be requested. The complete set of annotations available is detailed in the Supplementary Table S2.

After submitting the query, SNPnexus pre-processes the set of variants and shows a summary of the input with a basic set of annotations, namely mapping to known dbSNP, global population frequency, contig position and cytogenetic band. The input query is then submitted to the processing queue, with results presented in the renovated user interface. The user can download the results as text files or VCF files. Novel interactive visualizations are produced for appropriate annotations summarizing the results for easy inspection.

### New architecture

**Figure 1.**
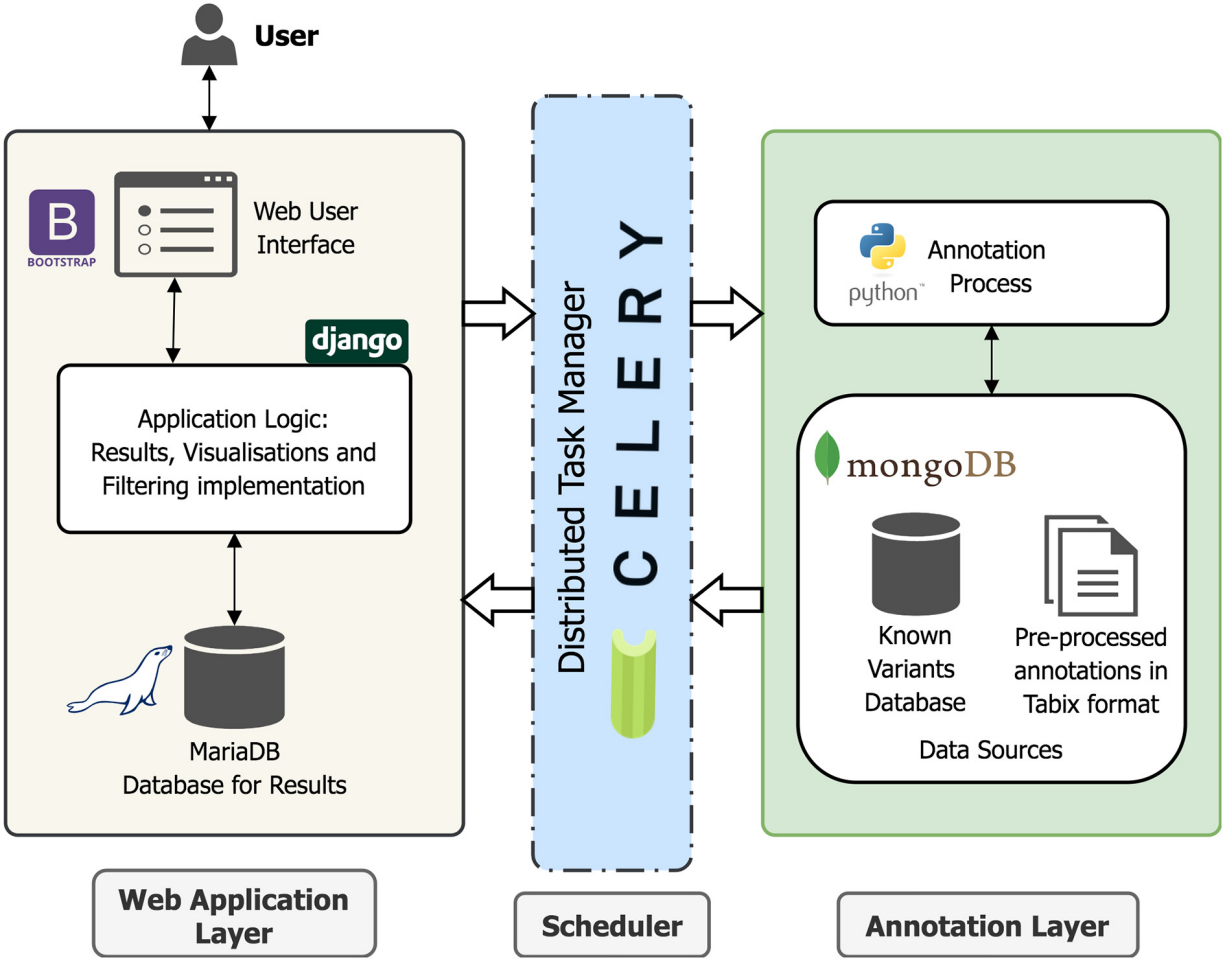
Schematic overview of the SNPnexus architecture. This version comprises a three-tiered framework: The Web Application Layer that deals with interactions with the user (submitting a query, showing results, generating visualizations and the filtering system); the Scheduler that acts as a communication interface and as a load balancer for the server; and the Annotation Layer that performs the annotation process on the input query set.

For this new release, SNPnexus has undergone a complete redesign both in its internal architecture and in its user interface. Even though SNPnexus has been designed to be used without any programming expertise, it is worth mentioning a few lines about the renewed architecture: the classic two-layered architecture of the previous releases has been replaced by three layers in which the web interface has been decoupled from the annotation process with a task manager working as a communication layer between them. A schematic overview of the new architecture is presented in Figure 1.

The first layer, the user interface, is a Django/Python application that deals with the user's requests and implements the results interface and the new filtering system. Once the input set has been validated, the second layer (Celery-based scheduler) sends the task into the queue and schedules the annotation process to run once the resources are available on the server. The task manager is also responsible for notifying the web interface once the annotations are completed. The third layer is the core of the annotation pipeline: a set of scripts that are capable of running in parallel with shared access to the data sources.

### New annotation categories and updated data sources

All the data sources for GRCh37 and GRCh38 human assemblies have been updated. This new version deprecates the old hg18 human assembly although this is still available from our legacy page (https://www.snp-nexus.org/legacy).

Most of the data sources have been pre-processed and converted to BED format that allows for the use of Tabix (9) for a fast query of data based on chromosomal position; many annotations for known dbSNPs have been pre-computed and stored on a MongoDB (10) database; and some annotations, namely the DeepSEA (11) scoring algorithm for non-coding variants and the Cancer Genome Interpreter, are installed in the server and executed when required for the input set. The complete list of data sources for this version of SNPnexus is also detailed in the Supplementary Table S2.

In order to bridge the gap between variant annotation and biological interpretation, two new annotation categories have been incorporated: Reactome Pathways (12) and the Cancer Genome Interpreter.

#### Reactome pathways

**Figure 2.**
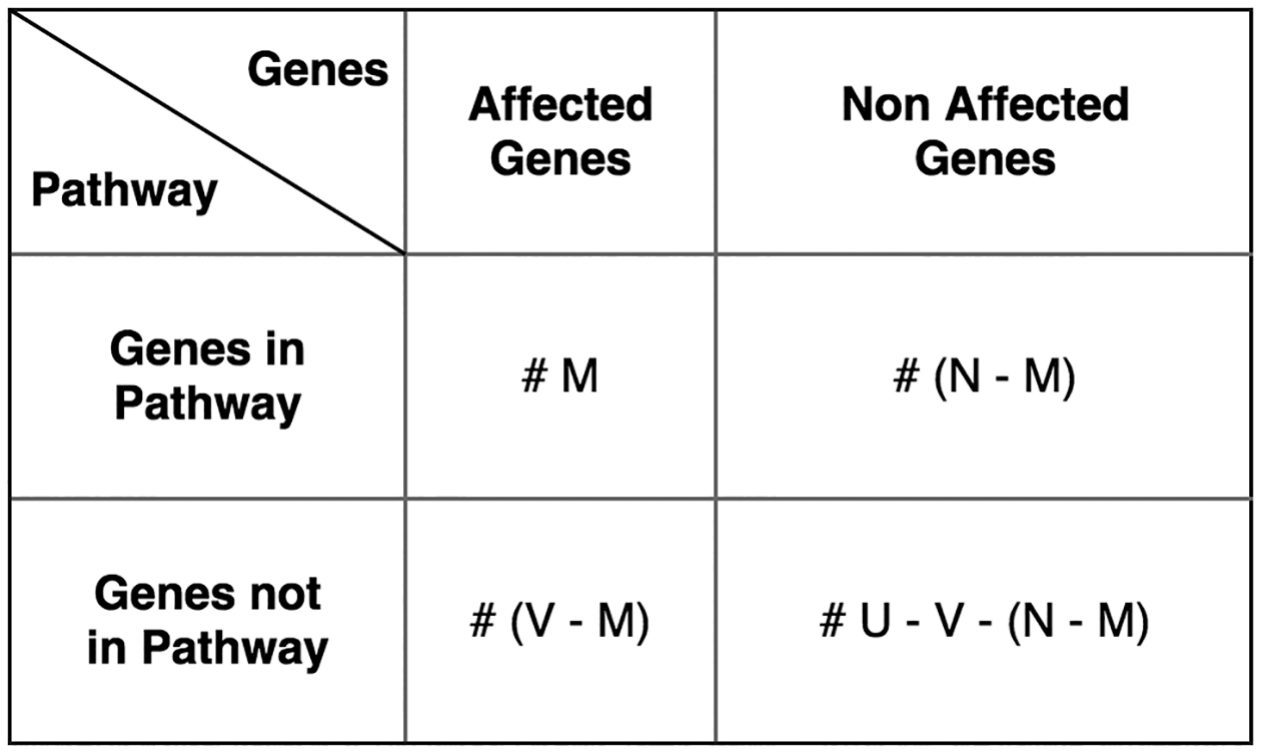
Contingency table for enrichment analysis. *N* = Number of affected genes; *M* (≤*N*) = affected genes in the pathway; *V* = number of genes in the Reactome pathway; *U* = number of genes in the Reactome universe.

**Figure 3.**
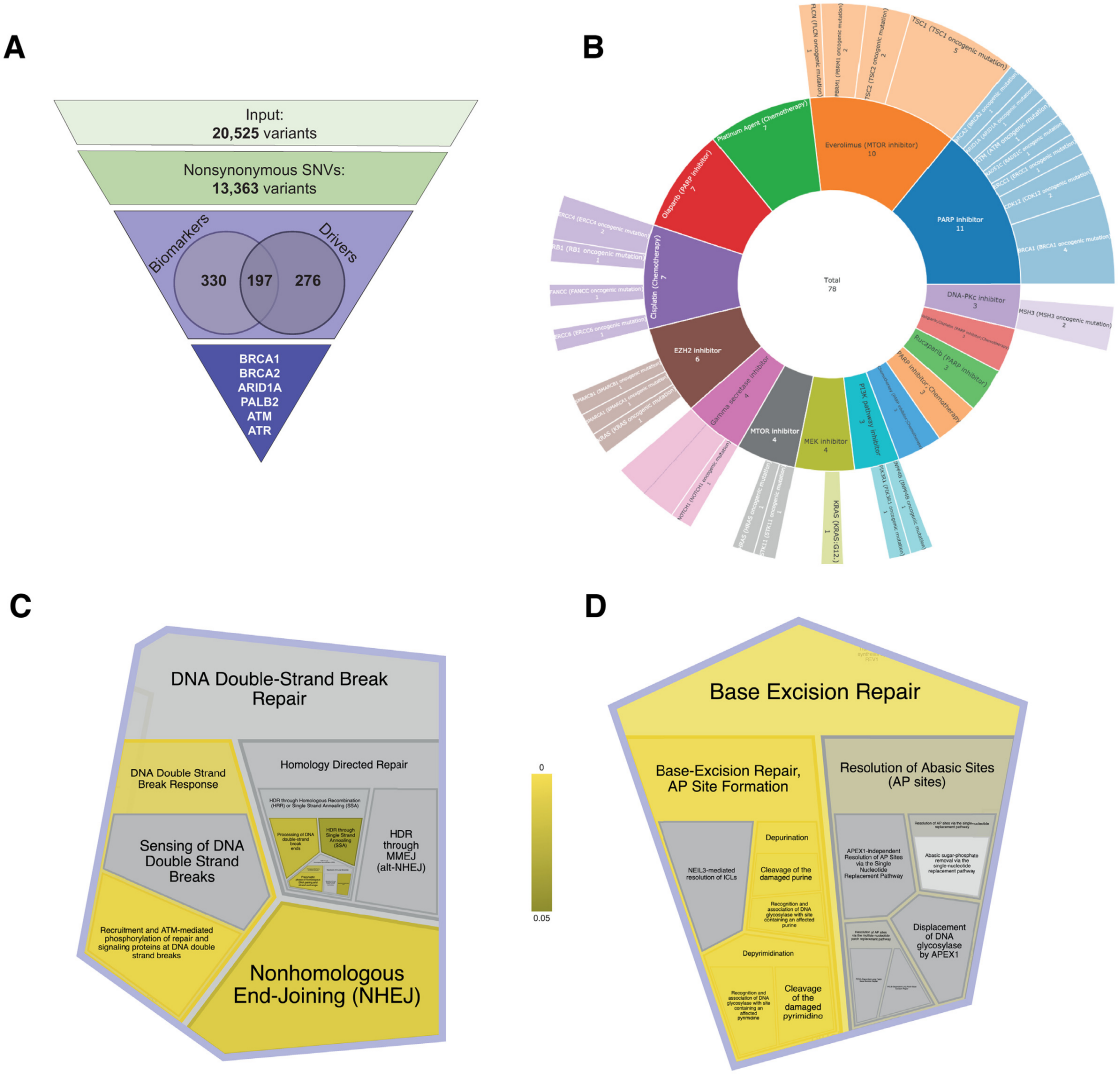
Analysis of sequence variations from TCGA-IB-7651. (**A**) Variant filtering method applied for the analysis of queried variants. Nonsynonymous SNVs were identified and prioritized according to their oncogenic classifications and targetable potential. (**B**) Several nonsynonymous variants were identified as potential biomarkers of response to targeted therapies for PDAC, including PARP inhibition and platinum-based chemotherapies. Pathways analyses of these variants identified enrichments within DNA damage repair (DDR) processes, including (**C**) DNA double-strand break response and (**D**) base excision repair. Please, refer to https://www.snp-nexus.org/v4/results/nar2020/ to have a better look of these figures and explore the results for the Case Study.

For each query set, SNPnexus links genes reported in each dataset to their associated biological pathway(s) (Reactome). First, the variants reported in each dataset are mapped to their corresponding gene. Next, using our MySQL instance of data obtained from Reactome (August 2019), we determine the number of genes within the Reactome universe, the number of genes in each pathway and the identity of genes linked to each pathway. These covariates are used to implement the Fisher's Exact method and determine whether the number of genes affected in each dataset is larger than expected by random chance for each Reactome pathway (Figure 2). Using this method, pathways over-represented within a gene list can be determined by considering both the number of affected genes within the user-defined gene list and the overall number of genes annotated within each Reactome pathway of interest. Analyses of overrepresented signaling pathways can provide useful insights into underlying biological and molecular processes, which can be used to identify genotype-phenotype interactions and inform on mechanisms of disease. Following the determination of statistically-enriched pathways, these results are provided in a tabular format as well as a link to an interactive Voronoi diagram, developed using ReacFoam (12). These visualizations highlight all pathways that are significantly enriched (*P* ≤ 0.05) in a yellow color scale. The interactive functions allow users to further examine the results at the individual-pathway level, as well as migrate through pathway hierarchies to determine biological processes affected at the uppermost hierarchical levels (e.g. Base Excision Repair → Base-Excision Repair, AP Site Formation → Depurination; Figure 3).

#### Cancer genome interpreter (CGI)

The CGI is a third-party tool developed to help in the interpretation of sequenced cancer genomes, assessing the potential of somatic alterations to act as tumor drivers and the possible effect on treatment response. This allows SNPnexus to highlight possible cancer driver mutations from its input query set and detect those that may be therapeutically actionable. As the CGI is only available at the moment for the GRCh37 human assembly, SNPnexus uses the liftOver tool (13) to make it available for GRCh38 as well.

### Improved interface, new visualizations and filtering system

The user interface has been completely redesigned, giving SNPnexus a more friendly and dynamic response to different screen sizes, resolutions and browsers. The query interface has been simplified and reorganized to make it more intuitive. A new processing page gives the user an initial set of annotations based on the genomic coordinates and also shows details of the status of the query.

The results page has been revamped by sorting the annotations by category and allowing the user to collapse or expand each category. Similarly, each category shows its set of annotations in a tabular interface. These changes make the results page less clutter and easier to navigate. As in previous versions, the results tables can be filtered, sorted and exported in VCF or tab-separated text formats.

A new set of interactive visualizations has been added. Karyotype plots for the genomic consequences and the predicted deleteriousness of the variants can be created. This plot allows the user to inspect the variants' positions in a view of the whole human chromosomes or focus on a specific chromosome.

The functional consequences of the variants can also be plotted in an interactive pie chart, with options to focus on the coding or non-coding variants. The predicted effect on proteins is also showed in a bar plot showing the number of tolerated or deleterious variants in the query set.

The Reactome Pathways are presented in an interactive Voronoi diagram format using ReacFoam. This format is ideal to visualize the pathways in their hierarchies and using the p-values as the color intensity, makes easier for the user to focus on the most interesting pathways affected by the variants query set. Only the pathways with p-value less than 0.05 are highlighted in this diagram (see Supplementary Figure S1).

A new sunburst chart for the biomarkers is presented. This shows in its first layer the drugs used in the trials and in its second layer the biomarkers affected by those drugs. This chart only shows biomarkers that match completely with the variants in the input query set.

The filtering system is the latest addition to the SNPnexus results page. Users are now able to tailor analyses and prioritize variations using this novel filtering functionality:

#### Type of variant

Users can filter the results to focus only on known SNPs or novel alterations.

#### Minor allele global frequency

It is possible to set a prevalence threshold for known variants. This filter uses data from gnomAD exomes, gnomAD genomes (14) and 1000 Genomes Project (15).

#### Gene

The results can be filtered to show only variants affecting one or more specified genes.

#### Genomic consequence

The user can filter the results to show variants based on the genomic consequences on up to 4 annotation systems for GRCh38 or up to seven annotation systems for GRCh37. The options available are Coding Nonsynonymous, Coding Synonymous, Intronic variants or variants on the UTRs zones.

#### Predicted effect

Using SIFT and PolyPhen scores, SNPnexus can filter predicted benign or damaging alterations.

#### Conserved region

SNPnexus can filter variants that lay within or outside a conserved region using data from Phast (16).

#### Phenotype association

It is possible to show only variants with a known phenotype association based on data from COSMIC and ClinVar.

#### Pathway

SNPnexus can show the results filtered from variants associated with one or more biological pathways specified.

#### Cancer driver

Users can filter the alterations based on their oncogenic classification, with options to show only the known and predicted mutations; or the polymorphisms from the CGI results.

## RESULTS

This new release enhances SNPnexus capabilities, providing not only an annotation tool but also, by using the new filtering system, a powerful and easy to use prioritization system that allows the user to highlight significant variants that may have a biological impact and focus on them for further studies.

The new architecture and the pre-processing of the data sources have improved the overall performance of the system especially notable in a drastic reduction of the annotation processing times. Table 2 shows a comparison of execution times between the previous version and the new release for different genomic annotations. It is worth mentioning that the improvement is more significant for large query sets.

**Table 2.** Comparison of computational speeds for a subset of annotations between the current and previous version of SNPnexus. Ensembl, RefSeq and COSMIC annotations are the most computationally intensive. Using an input file of 100k variants, SNPnexus now operates 16×, 15.5× and 82× faster than its predecessor for these annotations, respectively. SIFT is included to show that even for non-intensive annotations there is a noticeable improvement

		Processing time (s)	
Annotation	Number of variants	SNPnexus v.3	SNPnexus v.4
**Ensembl**	5k	172	7.36
	20k	2170	65.75
	50k	3909	158.19
	100k	7941	486.69
**RefSeq**	5k	137	4.28
	20k	1405	35.82
	50k	4265	347.31
	100k	7283	469.7
**SIFT**	5k	15	0.63
	20k	28	2.96
	50k	49	39.67
	100k	59	46.06
**COSMIC**	5k	2632	2.4
	20k	4623	10.46
	50k	8706	114.51
	100k	14279	173.53

The redesign of the user interface improves usability in all the stages of the annotation process, providing users with more information about their queries while they are being processed, and a clear results page better organized and with a new set of interactive visualizations. It is worth noting that these visualizations respect the filters applied by the filtering system.

The filtering system, included in the results page, provides a simple and innovative way to prioritize variants based on different parameters paving the way to identify alterations potentially associated with phenotypes. Furthermore, by adding the Cancer Genome Interpreter, SNPnexus may inform about variants that constitute biomarkers of response to anti-cancer drugs and the evidence that sustains this. Finally, the user guide and the examples of use have also been updated to reflect this new version.

### Case study

We tested the ability of SNPnexus to identify driver variants in cancer and prioritize targetable candidates for applications in precision medicine. Pancreatic ductal adenocarcinoma (PDAC) is projected to become the second leading cause of cancer mortality by 2030 (17). Surgical resection of early, localized disease provides the only chance for potentially curative treatment; however, most patients present with advanced disease and are not amenable for surgery. Chemotherapies, such as gemcitabine and the combination regimen FOLFIRINOX, are therefore regarded as the standard of care in patients who are diagnosed with surgically unresectable disease. These treatments provide little improvement to overall outcomes and offer only marginal survival benefits. Improving patient stratification towards targeted, molecularly-guided treatments has the potential to significantly improve outcomes in PDAC.

To demonstrate the utility of SNPnexus for the prioritization of targetable oncogenic mutations, we tested our tool using tumor sequencing data from a patient with PDAC. Sequence variations for patient TCGA-IB-7651 from the TCGA PAAD cohort were downloaded using TCGAbiolinks (18) and uploaded to SNPnexus as our query set (Figure 3). A total of 20,525 mutations from TCGA-IB-7651 were analyzed using SNPnexus. Genomic mapping and functional annotations of all queried variants were performed, and 13,363 coding nonsynonymous SNVs were identified. These results can be accessed, filtered and explored further from https://www.snp-nexus.org/v4/results/nar2020/.

For the analysis of cancer datasets, SNPnexus allows for variant filtering based on known tumor driver genes and the oncogenic potential of specific mutations. To prioritize variants bearing functionally significant consequences for driving tumor growth and progression, we filtered all nonsynonymous mutations by their oncogenic classifications, retaining those with a known/predicted driver status (Figure 3A). This identified a subset of 473 mutations, including variants within frequently mutated driver genes in PDAC, such as KRAS and SMAD4. Using the Reactome pathway analysis feature, we further identified enrichments for driver variants within several core PDAC signaling pathways (axon guidance, NOTCH signaling, RAS signaling) (19).

To investigate mutations relevant for targeted treatment, we filtered all coding variants for known biomarkers of therapeutic response and analyzed their corresponding biological pathways. Several potentially targetable alterations within the following genes involved in DNA damage repair (DDR) were identified: BRCA1, BRCA2, PALB2, ATM, ATR, RAD51C and ARID1A (Figure 3B). Previous pre-clinical and clinical studies have shown mutations within DDR genes to be potential targets for PARP inhibition or platinum-based therapies (20–26). SNPnexus identified enrichments for targetable alterations within DDR genes (Figure 3C) in TCGA-IB-7651, highlighting its utility for the identification of putative biomarkers for treatment response in patients.

The challenges in prioritizing functionally and clinically relevant variants from next-generation sequencing (NGS) data have hindered the identification of candidate molecular targets for both current and novel targeted therapies in PDAC. SNPnexus addresses the need for user-friendly, integrated annotation tools to streamline the downstream analysis and biological interpretation of variants derived from large NGS datasets, to guide precision medicine.

## DISCUSSION

With the explosion of genome variation data generated through high-throughput sequencing technologies, we have seen a rise in the number of specialized variant annotation tools, each designed to serve a specific purpose. SNPnexus has an established user base owing to its easily accessible web interface and a comprehensive set of annotation fields. There are several offline tools such as ANNOVAR (27), GEMINI (28) and Ensembl VEP (29) with similar strengths in terms of input size and annotation capabilities, but they need significant programming expertise and computational resources from the user. Comparisons with similar web-based variant annotation tools emphasize the user-friendly nature of SNPnexus—allowing for a range of data input and output formats to accommodate both single and batch queries, strengthened by the diversity of data annotation categories and data visualizations (see Supplementary Table S3). Web resources such as wANNOVAR (30) and Ensembl VEP Web Interface offer similar functionalities to SNPnexus but both fall short of the broad choice of annotations and visualizations available in SNPnexus. VarSome (31) is a powerful aggregator and impact analysis tool, but only allows for the exploration of a single variant, gene or genomic region at any one time. The broad choice of annotation fields provided by SNPnexus ensures that it remains relevant to the analysis and interpretation of sequence variants for a diverse range of biological applications.

Although SNPnexus accommodates variant analysis for general purpose, we acknowledge that variant annotation remains particularly pertinent within the growing field of cancer genetics. The inclusion of driver variant annotations and therapeutic biomarker predictions from the CGI allows users to identify and effectively prioritize phenotypically-relevant target mutations. Combined analysis of these predictions with the additional annotation fields provided by SNPnexus allows users to place candidate mutations within the broader biological context of each sequence dataset. The integration of Reactome pathway enrichment analyses further allows for investigations into the full complement of mutations within a tumor sample (driver and passenger variants), for the determination of affected oncogenic pathways; a feature which further distinguishes SNPnexus from other similar tools.

The new computational architecture of SNPnexus allows expanding processing capabilities but is limited by the hardware infrastructure. With potentially hundreds of requests to be processed concurrently, SNPnexus currently limits the maximum number of variants per query to 100 000. Only a handful of freely available web applications, such as Bystro (32) and DeepSea, make variant annotation and filtering possible for sequencing data on a whole-genome scale. However, DeepSea is particularly focused on the epigenetic state of a sequence to predict the functional impact of non-coding variations, whereas Bystro offers limited annotation categories and is more useful for pre-processing sequencing data to extract smaller variants datasets of interest. To the best of our knowledge, RegulationSpotter (33) is the only tool that offers comprehensive analyses for variants in both extragenic and intragenic (assessed by MutationTaster (34)) regions at the whole-genome scale with a human-readable simplistic biological interpretation. Tools like SNPnexus with advanced visualizations and additional annotation features, particularly pathway enrichment analysis or custom analysis of cancer-specific variants, can be efficiently used for further downstream applications and facilitate a comprehensive insight into individual mutations within each query set.

For future updates, we plan to release a command-line version of the tool, which will allow more experienced users to annotate any number of variants in their local infrastructure. Another feature will be the inclusion of patient cohort analysis. By allowing users to upload variants from multiple samples, VCF files and combining the annotation and filtering system, we plan to provide a novel way of identifying cohort common variants that are potentially interesting. Moreover, by continuing to expand our annotation categories, we envision further improvement of the biological and phenotypical interpretation capabilities of the tool.

## Supplementary Material

gkaa420_Supplemental_Files
