# Supplementary figures and images for: SNPnexus: a web server for functional annotation of human genome sequence variation (2020 update)
Source: Nucleic Acids Res. 2020 Jun 4;48(W1):W185–92. doi: 10.1093/nar/gkaa420 (PMC7319579; doi:10.1093/nar/gkaa420)

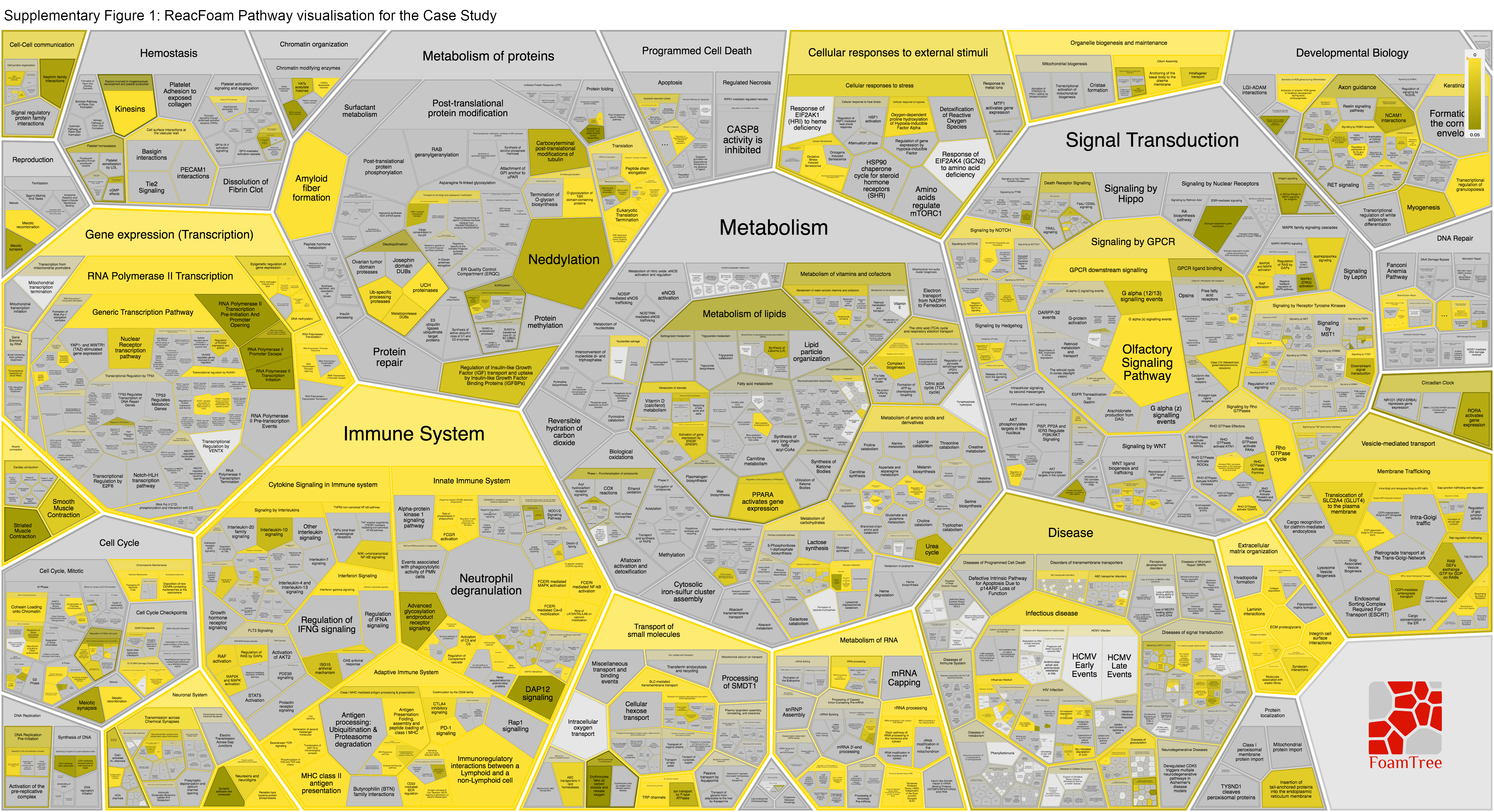

Supplement: gkaa420_Supplemental_Files [file gkaa420_supplemental_files.zip › Supplementary_Fig01_SNPnexus.png]
